# Therapeutic strategies to address neuronal nitric oxide synthase deficiency and the loss of nitric oxide bioavailability in Duchenne Muscular Dystrophy

**DOI:** 10.1186/s13023-017-0652-y

**Published:** 2017-05-25

**Authors:** Cara A. Timpani, Alan Hayes, Emma Rybalka

**Affiliations:** 1 College of Health & Biomedicine, Victoria University, PO Box 14428, Melbourne, Victoria Australia 8001; 2 Institute of Sport, Exercise & Active Living (ISEAL), Victoria University, Melbourne, Victoria 8001 Australia; 3 Australian Institute for Musculoskeletal Science (AIMSS), Melbourne, Victoria 3021 Australia

**Keywords:** Duchenne muscular dystrophy, Neuronal nitric oxide synthase, Nitric oxide, Skeletal muscle, *mdx* mouse, Clinical trials

## Abstract

Duchenne Muscular Dystrophy is a rare and fatal neuromuscular disease in which the absence of dystrophin from the muscle membrane induces a secondary loss of neuronal nitric oxide synthase and the muscles capacity for endogenous nitric oxide synthesis. Since nitric oxide is a potent regulator of skeletal muscle metabolism, mass, function and regeneration, the loss of nitric oxide bioavailability is likely a key contributor to the chronic pathological wasting evident in Duchenne Muscular Dystrophy. As such, various therapeutic interventions to re-establish either the neuronal nitric oxide synthase protein deficit or the consequential loss of nitric oxide synthesis and bioavailability have been investigated in both animal models of Duchenne Muscular Dystrophy and in human clinical trials. Notably, the efficacy of these interventions are varied and not always translatable from animal model to human patients, highlighting a complex interplay of factors which determine the downstream modulatory effects of nitric oxide. We review these studies herein.

## Background

Duchenne Muscular Dystrophy (DMD) is a progressive and fatal X-linked [1] neuromuscular disorder afflicting 1 in 3500–5000 live male births [2]. DMD arises from the loss of dystrophin [3], a 427 kDa cytoskeletal protein [4] that links the contractile apparatus to the sarcolemma via the dystrophin-associated protein complex (DPC). Dystrophin is believed to provide stability and integrity to the muscle membrane during contraction and in its absence, skeletal muscle is prone to damage. The alterations to the membrane induced by dystrophin-deficiency leads to an excessive influx of calcium (Ca^2+^) from the extracellular environment, which is poorly buffered, and activates Ca^2+^-dependent proteases to induce a cascade of degeneration and damage. As the disease progresses, and damage and degeneration accrues, the regenerative capacity of the muscle diminishes and becomes unable to match the demand for repair [5]. Muscle is subsequently replaced with fibrous and/or fatty connective tissue. Clinically, the increasing presence of non-functional muscle leads to muscle weakness and loss of function, with DMD sufferers wheelchair bound by early adolescence and eventually succumbing to cardiorespiratory failure by the third decade of life [6].

It is most commonly accepted that the excessive influx of Ca^2+^ into dystrophin-deficient myofibres is the catalyst for dystrophinopathy. However, emerging evidence suggests that metabolic and mitochondrial dysfunction may play a significant role in disease progression [7–9]. Whether this dysfunction is a secondary consequence to dystrophin-deficiency or independent is unknown, however a physical link between dystrophin and metabolism exists in neuronal nitric oxide synthase (nNOS). nNOS is an enzyme usually localised to the sarcolemma attached to the DPC, however in the absence of dystrophin, there is a secondary reduction of nNOS [10, 11]. The loss of nNOS from the sarcolemma reduces overall nNOS content in dystrophic muscle [12–15] resulting in decreased nNOS activity [12–15] and NO production [16–18]. The loss of nNOS protein and subsequently NO production capacity and bioavailability, is detrimental to dystrophic muscle for two reasons. Firstly, NO is an important signalling molecule involved in many biological processes including metabolism, blood flow and regulation of muscle function and mass [19]. Secondly, the nNOS protein itself interacts with phosphofructokinase (PFK), a regulatory enzyme of glycolysis, and is capable of increasing its activity by 60-fold [20] thereby increasing glycolytic rate and capacity. The loss of association between nNOS and PFK in dystrophin-deficient muscle may help to explain the fatigability of dystrophic muscle [21, 22] and may partially or fully account for the various glycolytic impairments observed [20, 23, 24]. In addition to the vast deficits in mitochondrial function (for detailed reviewed see [9]), these metabolic impairments reduce energy production capacity [7] and resting energy content [25, 26] which severely limits the muscles capacity to buffer damage and facilitate repair. As it appears that NO plays an important role in metabolism and the maintenance of skeletal muscle mass, restoring NO bioavailability in dystrophin-deficient muscle may be beneficial (summarised in Table 1). Here, we review the various approaches to restore NO bioavailability in dystrophic muscle including nNOS overexpression, ˪-arginine administration, phosphodiesterase (PDE) inhibition and nitrate supplementation, with a focus on the effects on the architecture, function and metabolism of dystrophin-deficient skeletal muscle.

**Table 1:** Summary of methods utilised to increase NO production and the effects observed in dystrophic skeletal and cardiac muscle from DMD animal models and patients

Method/Mechanism	Dosage Range	Model	Effects	Other	Reference
nNOS restoration *Breeding with transgenic nNOS overexpressors* *Transfection with nNOS*	N/A	*mdx* mouse Dystrophin/utrophin knockout mouse *mdx* mouse	*Skeletal muscle:* reduces inflammation, macrophage and neutrophil infiltration, damage *Cardiac muscle:* reduces fibrosis, macrophage infiltration, improves impulse conduction *Skeletal muscle:* increases DPC expression, NO production, reduces damage and fatigue, prevents force production loss		[39–45, 47–49]
˪-arginine supplementation	200–1000 mg/kg/day	DMD patients *mdx* mouse	*Skeletal muscle:* increases DPC expression, reduces damage, fibrotic and fatty tissue infiltration, inflammatory cell infiltration, oxidative stress, improves grip strength, contractile function and reduces fatigability	Administered in combination with metformin and prednisone	[18, 29–36]
PDE inhibition *Sildenafil* *Tadalafil*	0.7–80 mg/kg/day 30–300 mg/kg/day	DMD patients *mdx* mouse DMD patients *mdx* mouse	*Skeletal muscle:* reduces collagen and inflammatory cell infiltration, improves sarcolemmal integrity *Cardiac muscle:* reduces membrane permeability, induces cardiac remodelling, improves heart function *Skeletal muscle:* improves functional ischemia, reduces contraction-induced damage, fibrotic infiltration, histological variability, improves exercise performance, increases expression of ETC. genes		[52, 55, 57–61]
NO donation	21–80 mg/kg/day	*mdx* mouse	*Skeletal muscle:* increases vascularisation, blood flow, exercise performance and strength, decreases free Ca^2+^ concentration, damage, inflammation, fibrotic and collagenous infiltration *Cardiac muscle:* decreases damage, inflammation, fibrotic and collagenous infiltration, improves cardiac function and architecture	Administered in combination with NSAIDs	[62–69]
Expansion of nitrate-nitrite-NO pool	85 mg/L	*mdx* mouse	*Skeletal muscle:* does not improve mitochondrial deficits, increases damage and peroxynitrite production	Only one study to date	[107]

### Increasing nNOS substrate availability

NO is an important signalling molecule that elicits a myriad of physiological effects through the production of cyclic guanosine monophosphate (cGMP) and/or S-nitrosylation of thiol residues of cysteine groups. cGMP is a second messenger produced by the binding of NO to the enzymatic receptor soluble guanylyl cyclase (sGC) [27]. The increase in cytoplasmic cGMP activates downstream cGMP specific protein kinases, cation channels and PDEs which then exert various biological effects [27]. NO also mediates its effects through S-nitrosylation, a post-translational modification of proteins that modulates enzyme activity, protein stability and localisation [28]. Since the secondary dissociation of nNOS from the sarcolemma in dystrophic skeletal muscle reduces NO bioavailability, which would impair a multitude of physiological processes that may contribute to disease progression, various techniques to increase NO production have been investigated.

**Fig. 1:**
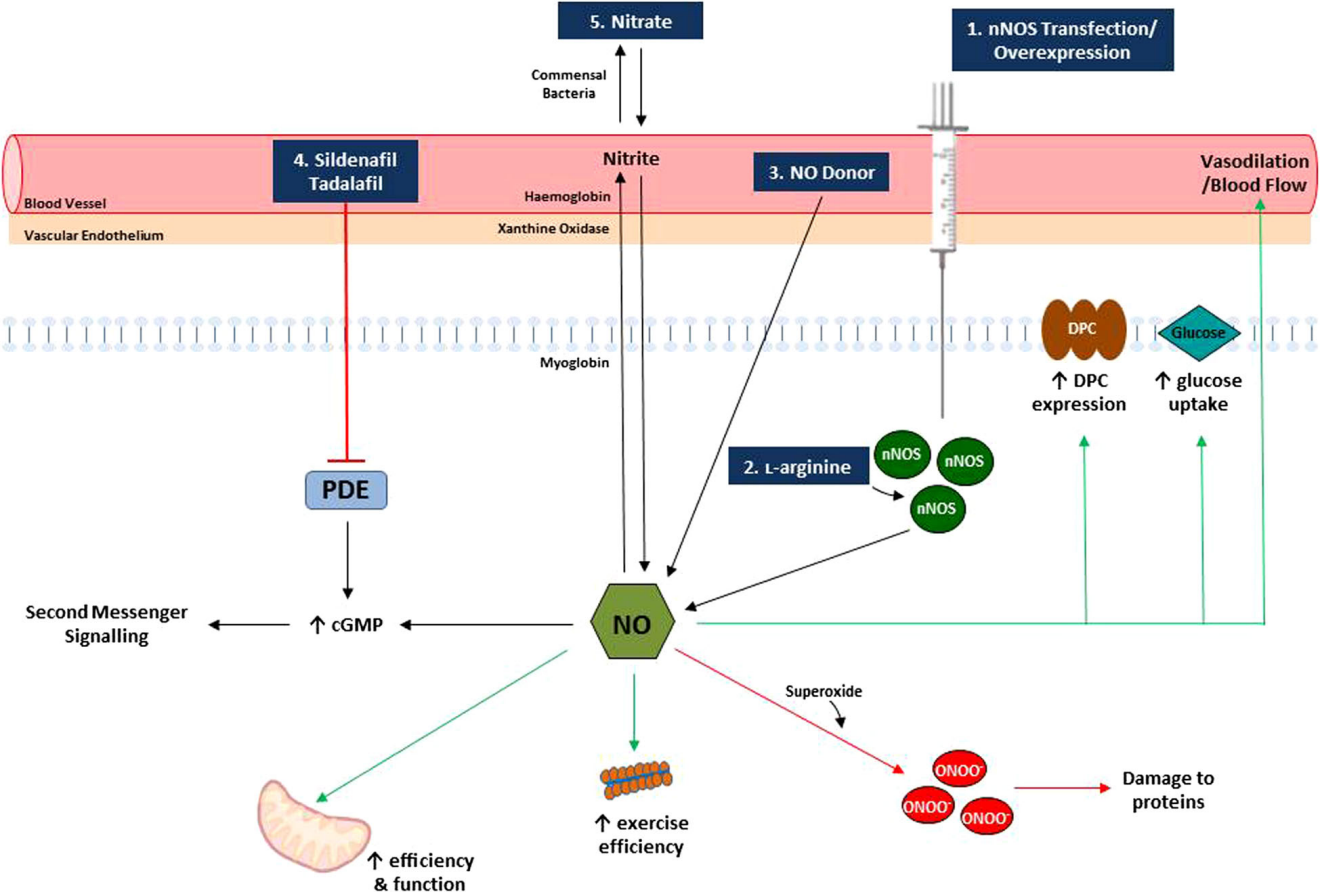
Schematic of methods utilised to increase NO bioavailability in dystrophic skeletal muscle and the downstream effects. Increasing NO bioavailability through (1) restoration of nNOS, (2) ˪-arginine supplementation, (3) NO donation and (4) inhibition of the enzyme phosphodiesterase (PDE) has led to increases in mitochondrial function, exercise capacity and stabilisation of the membrane in dystrophin-deficient skeletal muscle. A potential consequence of increased NO bioavailability, as observed through nitrate supplementation (5), is peroxynitrite (ONOO^−^) formation which can lead to further muscle damage and is undesirable in dystrophic skeletal muscle

Considering that nNOS delocalisation from the sarcolemma does not completely obliterate the nNOS protein in dystrophic skeletal muscle [11], substrate availability, in the form of ˪-arginine, may be a limiting factor to nNOS-dependent NO production (Fig. 1). ˪-arginine (50–100 mg/kg) administration in the *mdx* mouse demonstrably improves sarcolemmal integrity as indicated by increased utrophin – a dystrophin analogue – [18, 29–33] and DPC protein expression [18, 30–33], reduced Evans Blue Dye (EBD) uptake – a marker of skeletal muscle membrane damage – [18, 32–34] and decreased serum creatine kinase levels – a clinical marker of muscle damage and disease progression [30, 32, 33]. In a pilot trial involving 5 DMD patients, the combination of ˪-arginine (7.5 g/day) and the pharmacological Adenosine Monophosphate-activated Protein Kinase (AMPK)-activator, metformin (500 mg/day), decreased resting energy expenditure, shifted energy metabolism substrate preference to fatty acids, reduced oxidative stress and improved motor function [35]. NO is a known activator of AMPK, highlighting that promoting both the production of NO (i.e., with _L_-arginine) and the downstream metabolic responses that are normally modulated by NO (i.e., with metformin) can functionally improve the metabolism and function of dystrophic skeletal muscle. In a subsequent single-centre, randomised, placebo controlled-trial, aimed at recruiting 40–50 DMD patients, the same group is currently investigating the efficacy of combined ʟ-citrulline and metformin (NCT01995032; [36]) – ʟ-citrulline was chosen for this trial it is an ʟ-arginine precursor that can demonstrably restore muscular ʟ-arginine levels and reduce muscle wasting in ʟ-arginine-deficient conditions, while having a self-mediated effect on protein metabolism via inducible (i)NOS which bypasses the obvious nNOS deficiency [37]. ʟ-arginine therapy has also proven beneficial to skeletal muscle in the *mdx* mouse (a genetically homologous murine model of DMD). Histologically, ˪-arginine therapy improves many of the characteristic myopathological hallmarks in *mdx* mice including reductions in fatty and fibrotic tissue and collagen deposition [30–32, 38], inflammatory cell infiltration [31] and necrosis [18, 30, 32]. Functional improvements in grip strength [32, 33], lesser decrement in strength with age [38], and improved respiratory function [30, 32] were also observed. In addition to these functional improvements, ˪-arginine demonstrably reduces dystrophic muscle fatigability [33] and improves contractile function [30, 32] resulting in an increased capacity to exercise [34]. Whilst ˪-arginine administration appears to be beneficial both in the *mdx* mouse and DMD patients, the significantly reduced nNOS content evident in DMD patients suggests that there is a limited therapeutic application for ˪-arginine unless concomitant increases in nNOS expression could be achieved, or alternative isoforms of NOS could be exploited (i.e. through iNOS as per ʟ-citrulline therapy). This is especially true since ˪-arginine administration alone, especially in high doses, can have adverse side effects [39]. Indeed, a recent paper describing metabolic biomarkers of DMD demonstrates significantly elevated serum arginine concentrations in DMD patients as the disease progresses [40], highlighting the possibility of an ineffective uptake either alone or in combination with an ineffective metabolism due to reduced nNOS protein at the skeletal muscle level. While reduced ʟ-arginine transporter protein expression has been demonstrated in cardiac muscle from two murine models of DMD (*mdx* and *mdx*/*utrophin* double knockdown mice) [41], there is no data describing this deficit in skeletal muscle either in mice or in human DMD patients. Such a deficit would logically explain a reduced capacity for ʟ-arginine uptake resulting in plasma accumulation, and may be a consequence of a reduced capacity for metabolism by nNOS.

### Restoring nNOS protein expression

Given that the limiting factor to nNOS substrate supplementation therapy would be sufficient nNOS to catalyse the NO-generating reaction, restoring nNOS protein, particularly to the sarcolemma, presents as a strong therapeutic candidate. Indeed, restoring nNOS levels in dystrophic skeletal muscle has proven to be beneficial (Fig. 1). Offspring of transgenic nNOS overexpressors bred with the *mdx* mouse show a significant mitigation of membrane damage as reflected by a reduction in inflammation, macrophage and neutrophil infiltration, centronucleation of fibres and membrane lesions [42, 43]. Introduction of this nNOS transgene also extends protective effects to the dystrophic heart by reducing fibrosis and macrophage infiltration in conjunction with improving impulse conduction [44]; and to the neuromuscular junction through improvements in neuromuscular junction size and architecture in the presence of α-syntrophin [45]. Remarkably, nNOS restoration in *dystrophin/utrophin* knockout mice (which phenotypically resemble DMD) increases survival rate while reducing macrophage infiltration and the fibrotic and connective tissue content of dystrophin deficient skeletal muscle [46]. Therefore, increased expression of nNOS has a protective effect on maintaining muscle architecture and preventing membrane lysis through the normalisation of NO production [47]. Moreover, transfection with a modified muscle specific nNOSμ isoform – which localises to the membrane without the presence of dystrophin – resulted in increased expression of utrophin and other DPC proteins (including α-syntrophin and β-dystroglycan) which induced localised NO production at the sarcolemma and protection against contraction-induced damage and fatigue [48]. In contrast, an unmodified nNOSμ afforded less protection than the modified muscle-specific nNOSμ isoform in the same study [48]. This appears to be reflective of the binding of the modified NOS to the membrane via palmitoylation which induced utrophin expression. While the mechanism as to why membrane-localised nNOS induces utrophin expression is unclear. This utrophin upregulation seems to protect dystrophic *mdx* muscle from progressive damage, particularly as *mdx* mice age [49], thus attenuating the dystrophic phenotype despite the absence of dystrophin expression. Insertion of a mini-dystrophin gene via a dual adeno-associated viral vector which increases mini-dystrophin expression and restores nNOS at the sarcolemma [50], has also been shown to improve contraction-induced ischemia and mitigate the loss of force production and muscle damage [51, 52]. Collectively, these data highlight that increased expression of the nNOS protein, irrespective of localisation within the cell, can improve various characteristics of the dystrophic condition. However, there may be limited long-term therapeutic potential for nNOS overexpression as a delocalised nNOS (from the sarcolemmal DPC) becomes a substrate of calpains [53]. Calpains are enzymes that stimulate protein damage and are particularly active in DMD pathology [54]. Thus increased calpain activity may significantly reduce unbound nNOS expression and induce further non-specific protein damage. Since nNOS deficiency has also been documented to increase ryanodine receptor-mediated Ca^2+^ leak [55], which would perpetuate Ca^2+^-dependent calpain activity, these data indicate that there is necessity in dual upregulation of nNOS and dystrophin to minimise unbound nNOS as a target for calpains which would promote the disease phenotype.

### Inhibition of phosphodiesterase activity

Given that enhancing NO production capacity is beneficial in dystrophic muscle yet there are complexities associated with re-insertion/establishment of dystrophin and nNOS expression, other mechanisms to increase NO bioavailability have been investigated. One such avenue is the inhibition of the PDE family which breakdown phosphodiester bonds in second messenger molecules [56]. Specific PDEs hydrolyse cGMP thereby degrading it and decreasing cGMP second messenger capacity [56]. Since NO activates cGMP cycling, and it’s production and bioavailability is reduced in dystrophic muscle, pharmacologically prolonging/amplifying the cGMP signal would have likely benefits in NO-deficient cells (Fig. 1).

Inhibition of PDE5A has been commonly investigated in the *mdx* mouse as PDE5A is present not only in vascular smooth muscle [57], but also skeletal muscle [58] and to a lesser extent cardiac muscle [59], thereby allowing for a systemic effect of a prolonged NO signal in these tissues. Treatment with Tadalafil (1 mg/100 mL), a pharmacological PDE5A inhibitor, has shown to be beneficial in overcoming functional ischaemia following contraction, which was partnered with reduced contraction-induced sarcolemmal damage and muscle fibre death [60]. Tadalafil treated *mdx* muscles (30 mg/kg/day) also demonstrated histological improvements with a decrease in EBD uptake, fibrotic infiltration, centronucleated fibres and fibre size variability [60, 61] suggesting less damage and prevention of muscle degeneration. Additionally, exercise-induced damage was minimised in Tadalafil treated mice as evidenced by reduced Ca^2+^ accumulation [61]. Functionally, time to exhaustion from treadmill running and extensor digitorum longus (EDL) strength were concomitantly improved following Tadalafil treatment [61] in addition to post-exercise increases in activity and reductions in serum creatine kinase and muscle oedema [62]. PGC-1α expression was also increased following Tadalafil treatment alongside an enhanced expression of various electron transport chain genes suggestive of a fibre type shift to an oxidative phenotype [61]. Considering the vast mitochondrial and oxidative metabolism deficiencies observed in dystrophic muscle [9], upregulation of mitochondrial and oxidative genes would likely be beneficial to dystrophic muscle. Only two studies to date have documented the successful translation of Tadalafil treatment into small populations of DMD [63] and Becker Muscular Dystrophy (BMD) [64] patients. Although these trials were small, and primarily assessed improvements in functional muscle ischaemia as an endpoint measure, these results demonstrate that the beneficial effects of PDE5A inhibitors in pre-clinical studies are translatable in patients with dystrophin-deficiency. In particular, both of these studies utilised an acute treatment protocol (up to 2 days) with endpoint measures assessed an hour following treatment indicating an effect of PDE5A inhibition in these populations. A recent study by Hammers et al. [65] has also demonstrated a cardioprotective role for Tadalafil whereby a daily dosage of 1 mg/kg for 16 months reduced dystrophy-related histopathological features, calpain-mediated proteolysis and preserved cardiac function (as assessed by echocardiography and MRI). A notable limitation to this study was the low number of animals utilised (*n* = 2 DMD and control).

Similar results have been observed with the alternative PDE5A inhibitor, Sildenafil. In *mdx* mice, Sildenafil demonstrably increases specific force, reduces collagen I, fibronectin and TNFα infiltration, and improves sarcolemmal integrity of the diaphragm [66]. However, these improvements did not result in changes to mitochondrial function nor improvements in ATP production as originally hypothesised [67]. Since DMD patients typically succumb to respiratory failure, these data importantly highlight the potential for Sildenafil to prolong the lifespan of DMD patients should diaphragmatic improvements be translatable in the clinical setting. Following phenotypic drug screening in dystrophic zebrafish, a PDE inhibitor (aminophylline), which has similar properties to Sildenafil, was shown to have the greatest capacity to restore normal skeletal muscle structure [68]. The group later demonstrated beneficial effects of Sildenafil in the *mdx* ^*5cv*^ mouse model via enhanced signalling of haemoxygenase and downstream cGMP [69]. Sildenafil also appears to induce protective effects in the *mdx* heart by reducing membrane permeability and altering the expression of proteins implicated in beneficial cardiac remodelling [70]. Functionally, Sildenafil normalises heart rate responses to increasing workload [70] and reverses ventricular dysfunction [71]. Again, since cardiac complications reduce lifespan in DMD patients, these data appear to be promising should they be translatable in the clinical setting.

Collectively, the studies investigating PDE inhibition suggest that amplification of the typically NO-dependent cGMP signal benefits both skeletal and cardiac function and mitigates various characteristics of the dystrophic condition in the *mdx* mouse, and in an acute setting, is beneficial in both DMD and BMD patients. Recently, however, a Phase 3 clinical trial of Sildenafil in DMD and BMD boys was prematurely stopped following the absence of improvements in skeletal muscle function and adverse changes to left ventricle volumes (NCT01168908; [72]). In a parallel Sildenafil trial in only BMD patients, the clinical trial was completed but no obvious benefit to patients was observed (NCT01350154; [73]). Most recently, a Phase 3 clinical trial in only DMD patients investigating the capacity of Tadalafil to slow the decline of ambulation was prematurely terminated due to lack of efficacy. These clinical data indicate that Tadalafil and Sildenafil are not translatable drugs from pre-clinical studies to patients with DMD when the patients receive treatment for a chronic period of time (i.e., 6 months) or are on standard of care (SoC) therapy. While the inhibition of PDE5A is pre-clinically viable (i.e., in mouse, zebrafish and dog models), it must be kept in mind that these preclinical studies were not performed in conjunction with SoC therapies (i.e., prednisone or deflazacort). From a pharmacological standpoint, both Tadalafil and Sildenafil are drugs marketed for a specific indication (Tadalafil for erectile dysfunction and benign prostatic hyperplasia, Sildenafil for erectile dysfunction and pulmonary arterial hypertension) that happened to find efficacy in other indications both pre-clinically and clinically. The hope of testing drugs like Tadalafil and Sildenafil are that they are already FDA approved, albeit for other indications, and have known safety margins. However, the recent discovery that BMD patients, who express a truncated version of dystrophin, are also deficient in PDE5A [73] suggests limitations to this therapeutic avenue. This deficiency highlights that as per nNOS, the expression of PDE is intimately linked with dystrophin and/or DPC expression, and that the capacity to exploit them pharmacotherapeutically is therefore limited in DMD. As such, no improvements in cardiac function, blood flow to the skeletal muscle during exercise, or quality of life were observed in BMD patients [73].

### NO donors

Since ʟ-arginine and PDE activation are both dependent upon the presence of key enzymes/proteins associated with the sarcolemma, and more specifically, the DPC, promoting NO production through the use of NO donors may be of greater benefit to bypass this defective/inefficient protein system. As there is limited nNOS present in dystrophin-deficient skeletal muscle, this significantly impairs the muscles capacity for NO production. Therefore, even with PDE inhibition, the availability of NO would still be significantly diminished. Thus, the use of NO donors is an attractive therapeutic treatment option as they have the capacity to markedly increase systemic NO availability beyond the capacity to endogenously produce it within dystrophic muscle (Fig. 1).

Indeed, 6 months delivery of a nitric ester derivative of sedative alkyl alcohol (administered at 40 mg/kg 5 days/week) has been shown to enhance the vascular density of skeletal muscle, as well as exercise performance and strength in *mdx* mice, with a marked decrease in the free intracellular Ca^2+^ concentration of skeletal muscle [74]. In addition, the NO-donating nitric ester increased muscle fibre size while concomitantly reducing the population of regenerating fibres, suggestive of decreased damage [74]. Similarly, 7 months of 30 mg/kg naproxcinod, a non-steroidal anti-inflammatory drug (NSAID) with NO-donating properties, in food, had a beneficial effect on the running capacity of *mdx* mice with both time to exhaustion and whole body strength improved [75]. These functional benefits were partnered with improved muscle architecture, and reductions in inflammatory, fibrotic and collagen infiltrate observed in both skeletal and cardiac muscle [75]. Longer term administration of naproxcinod (at 21 mg/kg/day in food for 9 months) induces similar improvements in the strength and histological properties of cardiac muscle leading to the functional normalisation of ejection fraction time, and systolic blood pressure [76]. Considering the anti-inflammatory effects of the aforementioned NO-donors, combining a NO donor with NSAIDs could enhance the beneficial effects of NO. Three months of a HCT 1026-enriched diet (NO donor derived from flurbiprofen; 45 mg/kg/day) significantly improved blood flow and alleviated functional ischaemia in *mdx* mice [77]. A longer term supplementation regimen of the same drug (30 mg/kg/day in food for 12 months) was shown to reduce muscular damage, with concomitant decreases in serum creatine kinase levels and improved mobility of *mdx* mice [78]. Moreover, the addition of isosorbide dinitrate (30 mg/kg/day) with ibuprofen (50 mg/kg/day) has been shown to induce significant protection of the dystrophic heart by normalising left ventricle mass and wall thickness, maintaining cardiomyocyte number and reducing cross sectional area. Reduced fibrotic tissue content and inflammatory cell infiltration and a concomitant improvement in overall cardiac function was also observed in the *mdx* mouse [79]. Isosorbide dinitrate, alone (66 mg/kg) or in combination with prednisone (1 mg/kg) for 18 days, also demonstrably improves sarcolemmal integrity, decreases the presence of calcified fibres and stimulates regeneration in the *mdx* diaphragm, however without the addition of ibuprofen, it promoted an increase in heart weight [80] which was not observed previously [79]. An increase in cardiac mass, without improvements in cardiac function, is considered an adverse effect of treatment which would promote the normal, progressive cardiac hypertrophy observed in DMD patients. Ibuprofen seems to abate this adverse effect since a safety study in DMD patients using 12 months of isosorbide dinitrate (40 mg/day) and ibuprofen (400 mg/day) maintained cardiac function and reduced systemic inflammatory markers [81]. Given there is the capacity for non-specific, systemic NO-donors to adversely affect cardiac tissue, the use of skeletal muscle targeted NO donation would be beneficial. Indeed, oral administration of MyoNovin (80 mg/kg) – a NO donor that specifically donates NO to skeletal muscle – for 18 days induces similar effects to isosorbide dinitrate in *mdx* mice without the adversity of cardiac hypertrophy induction [80]. As the majority of the investigated NO donor therapies have additional indications (i.e., as anti-inflammatories (ibuprofen) or muscle relaxants (MyoNovin)), it is difficult to separate out the benefits specifically provided by donated NO and it is possible that that these alternative indications may be the more pertinent effectors. Given that uncontrolled and excessive NO delivery can induce pathological effects including inflammation, mitochondrial dysfunction and myocardial damage [82], these data suggest that manipulation of the NO-donation delivery system may be pivotal to mitigating the unwanted side effects of NO donor therapy. As delivery of NO to the skeletal muscle is difficult to control with pharmacological NO donors, and needs to be highly regulated since changes in NO concentration can be either beneficial, deleterious or insignificant to the promotion of Ca^2+^dysregulation [83], availability of a constant yet buffered reserve of NO is important.

### Nitrate supplementation

Recently, it has emerged that dietary supplementation with nitrate increases endogenous NO production via a nNOS-independent pathway (Fig. 1). Nitrate is an inorganic anion that is abundant in green leafy vegetables including beetroot, lettuce and spinach [84] and also in carrot, beetroot and pomegranate juices [85]. The nitrate anion is inert but once ingested, nitrate is reduced by the commensal bacteria in the enterosalivary pathway [86] into the bioactive nitrite, which then circulates in the blood. Although bioactive, nitrite is further converted to NO via several enzymatic pathways in the blood and tissues, including xanthine oxidase, myoglobin and haemoglobin [87], to exert a range of physiological effects. Thus, this pathway is complementary to nNOS-derived NO production. Additionally, there is benefit to this nitrate-nitrite-NO pathway as it is reversible. NO can be oxidised back to nitrate by myoglobin and haemoglobin and therefore the capacity to cycle back to nitrate allows for a constant reservoir of NO [88]. Moreover, since chronic increases in NO bioavailability can be toxic and induce systemic pathology [82], having an inactive reservoir of buffered NO would be beneficial. Therefore, enhancing the nitrate-nitrite-NO pathway represents a potential pathway that could be exploited to significantly enhance NO availability in dystrophic muscle in a controlled and buffered manner.

Recent studies suggest that nitrate supplementation enhances health and skeletal muscle performance. A 3 day oral supplementation of sodium nitrate (0.1 mmol/kg/day) in healthy males revealed that nitrate significantly improved skeletal muscle mitochondrial bioenergetics by increasing mitochondrial efficiency and decreasing proton leak; and reduced whole body oxygen consumption following submaximal exercise [89]. Moreover, a 7 day supplementation regimen in drinking water of healthy mice (~3.75 μmol/day) significantly improved skeletal muscle contractility, particularly of the EDL, by increasing expression of Ca^2+^ handling proteins [90]. Similar improvements in contractile function have also been observed in humans following acute supplementation with nitrate-rich beetroot juice (0.6 g/300 ml), with the authors noting improved excitation-contraction coupling (at low frequencies) and increased explosive force production in quadriceps [91]. Acute beetroot supplementation also demonstrably reduces whole body oxygen consumption [92–95], promotes fatigue resistance [96–99] and improves performance times [97, 100, 101]. Similar data has been observed in rats using dietary sodium nitrate supplementation in drinking water (0.7 mM), which stimulated mitochondrial biogenesis (peroxisome proliferator-activated receptor β/δ and PGC-1α expression) and enhanced bioenergetics in both skeletal [102] and cardiac muscle [103] indicating that acute exposure to nitrate supplementation has a modulatory effects on bioenergetics.

The benefits of nitrate supplementation also extend to disease states. In chronic obstructive pulmonary disease patients, acute beetroot juice supplementation improved exercise capacity and decreased blood pressure [104, 105]. Similar findings – in addition to increased tissue oxygenation – were observed in peripheral artery disease patients [106]. Considering that dystrophic muscle is in a comparable metabolically-stressed state to exercising muscle in that there is an increased metabolic demand and sarcoplasmic [Ca^2+^]_,_ and that nitrate supplementation can elicit positive physiological responses in diseased tissue, investigating such a therapy for DMD is rational.

To date, we are the only group to have investigated nitrate supplementation in the *mdx* mouse and its downstream effects on muscle metabolism and architecture [107]. Previously, it was demonstrated that 8 weeks of 85 mg/L sodium nitrate in drinking water ameliorated metabolic syndrome in endothelial NOS-deficient mice by increased circulating plasma NO levels [108], suggesting that similar improvements could be observed in the *mdx* mouse through the restoration of NO availability. As metabolic dysfunction and insufficiency is a dominant feature of dystrophin-deficient muscle, we investigated two metabolic pathways that can be mediated by NO — glucose uptake and mitochondrial function. We demonstrated normal basal- and contraction-induced glucose uptake in *mdx* muscles, which is consistent with previous reports of normal insulin-dependent glucose uptake (which is NO-independent) in dystrophin-deficient muscle [109]. However, nitrate supplementation was unable to improve the depressed mitochondrial respiration observed in the white and red portions of the gastrocnemius in this study and as reported by us [7] and others previously ([8, 110, 111]. In fact, nitrate reduced the maximal respiration in the red gastrocnemius and failed to increase markers of mitochondrial biogenesis such as mitochondrial electron transport chain complex proteins. Our data is consistent with the recent observation that nuclear-specific NO production via localised nNOS is important to modulate nuclear-regulated mitochondrial biogenesis in skeletal muscle [112]. Thus, non-specific and unregulated NO generation by non-nNOS sources appears to be futile in the absence of nNOS expression and its regulatory function.

One benefit of nitrate supplementation in *mdx* mice that we did observe was reduced hydrogen peroxide generation, indicating reduced oxidative stress at the mitochondrial level [107]. Outright, the reduction in hydrogen peroxide appeared to be a positive effect; however, it occurred concomitant with a significant increase in reactive nitrogen species generation as determined by the immunolabelling of nitrotyrosine, an indirect marker of peroxynitrite. Peroxynitrite formation corresponded with increased damage of the tibialis anterior muscle as assessed by haematoxylin and eosin staining [113]. Our finding is in stark contrast to those who have previously demonstrated decreased muscle damage through NO donor therapy [78, 114], highlighting that the anti-inflammatory compounds often administered in combination with the NO donor in these studies, may be efficaciously offsetting the inflammatory response that can be induced by both NO and NO-induced peroxynitrite-mediated damage.

One clinical trial has also investigated sodium nitrate therapy in BMD patients. In this study, an acute single oral nitrate dose (140 mL beetroot juice concentrate containing 8.4 mmol inorganic nitrate) was shown to improve functional sympatholysis and post-exercise hyperaemia in ambulatory BMD patients [115]. While we did not measure these parameters in our study, it is most likely that the beneficial effects elicited by nitrate in BMD patients is reflective of the presence of both dystrophin and nNOS in their skeletal muscle, albeit these proteins are often severely, but not exclusively, reduced in BMD patients compared to healthy individuals [115]. It appears that there is a defined level of nNOS protein, or localisation of nNOS, that must be expressed in skeletal muscle to ensure that the normal and beneficial modulatory effects of bioavailable NO are exerted within the muscle. Indeed, this is apparent not only in our study but also in that of Nelson et al. [115] who reported several BMD patients that were non-responsive to nitrate therapy. This effect may be due to a more advanced disease/clinical state in these particular patients in which nNOS protein levels become reduced below threshold levels due to escalating dystropathology (i.e., protease activity).

## Conclusions

NO plays an important role in a variety of biological processes and in dystrophin-deficient muscle where NO production is limited due to the secondary reduction of nNOS, it is likely a significant contributor to disease progression. While improvements in muscle function, architecture and metabolism have been demonstrated using various methods to increase NO bioavailability including restoration of the nNOS protein, ˪-arginine supplementation and PDE inhibition, there are also limitations and/or side effects that need to be addressed. This is particularly true since the beneficial effects observed in pre-clinical animal models of DMD have largely failed to translate to clinical improvements in DMD patients. In the one ʟ-arginine supplementation study that has successfully translated in a clinical human DMD pilot trial, these patients were notably steroid naïve, and thus not receiving SoC treatment. These data highlight important drug/functional interactions that clearly require further elucidation. There are also variations in the outcomes at the muscle fibre level with different models of NO induction. For example, beneficial effects have been observed with NO donors, typically in addition with anti-inflammatories or which have additional alternate functions at the muscle level (i.e., as muscle relaxants as per MyoNovin), but not with nitrate supplementation. Moreover, it appears that chronic manipulation of the nitrate-nitrite-NO pathway may not be a viable therapeutic option for DMD given its tendency to promote damage and further dystropathology. While we are currently the only group to have investigated nitrate supplementation as a therapy for DMD, our findings suggest that long-term/chronic nitrate supplementation is detrimental to dystrophin-deficient muscle and may require a concomitant increase in nNOS protein expression to impart the same benefits it does in healthy muscle. This idea is supported by the beneficial effects seen in BMD patients following acute sodium nitrate supplementation, who express low, yet detectable, levels of both dystrophin and nNOS. However, further investigation is required to fully elucidate this intimate relationship. Targeting NO delivery to skeletal muscle with the concomitant induction of nNOS protein expression, appears to be a logical future direction in the utilisation of NO donation as a therapy for DMD. The translational capacity of this research however must address nNOS therapies in conjunction with SoC (i.e., prednisone, deflazacort), as this research is currently lacking in pre-clinical animal models.

## Abbreviations

AMPK: Adenosine monophosphate-activated protein kinase
BMD: Becker muscular dystrophy
Ca^2+^: Calcium
cGMP: Cyclic guanosine monophosphate
DMD: Duchenne muscular dystrophy
DPC: Dystrophin-associated protein complex
EBD: Evans blue dye
EDL: extensor digitorum longus
iNOS: Inducible neuronal nitric oxide synthase
nNOS: Neuronal nitric oxide synthase
NO: Nitric oxide
NSAID: Non-steroidal anti-inflammatory drug
ONOO^−^: Peroxynitrite
PDE: Phosphodiesterase
PFK: Phosphofructokinase
sGC: Soluble guanylyl cyclase
SoC: Standard of care

## References

[1] Monaco AP, Bertelson CJ, Middlesworth W, Colletti C-A, Aldridge J, Fischbeck KH, Bartlett R, Pericak-Vance MA, Roses AD, Kunkel LM (1985). Detection of deletions spanning the Duchenne muscular dystrophy locus using a tightly linked DNA segment. Nature.

[2] Emery A (1991). Population frequencies of inherited neuromuscular diseases--a world survey. Neuromuscul Disord.

[3] Hoffman EP, Brown RH, Kunkel LM (1987). Dystrophin: the protein product of the Duchenne muscular dystrophy locus. Cell.

[4] Koenig M, Monaco AP, Kunkel LM (1988). The complete sequence of dystrophin predicts a rod-shaped cytoskeletal protein. Cell.

[5] Heslop L, Morgan JE, Partridge TA (2000). Evidence for a myogenic stem cell that is exhausted in dystrophic muscle. J Cell Sci.

[6] Eagle M, Baudouin SV, Chandler C, Giddings DR, Bullock R, Bushby K (1967). Survival in Duchenne muscular dystrophy: improvements in life expectancy since 1967 and the impact of home nocturnal ventilation. Neuromuscul Disord.

[7] Rybalka E, Timpani CA, Cooke MB, Williams AD, Hayes A (2014). Defects in mitochondrial ATP synthesis in dystrophin-deficient Mdx skeletal muscles may be caused by complex I insufficiency. PloS one.

[8] Onopiuk M, Brutkowski W, Wierzbicka K, Wojciechowska S, Szczepanowska J, Fronk J, Lochmüller H, Górecki DC, Zabłocki K (2009). Mutation in dystrophin-encoding gene affects energy metabolism in mouse myoblasts. Biochem Biophys Res Commun.

[9] Timpani CA, Hayes A, Rybalka E (2015). Revisiting the dystrophin-ATP connection: how half a century of research still implicates mitochondrial dysfunction in duchenne muscular dystrophy aetiology. Med Hypotheses.

[10] Brenman JE, Chao DS, Xia H, Aldape K, Bredt DS (1995). Nitric oxide synthase complexed with dystrophin and absent from skeletal muscle sarcolemma in Duchenne muscular dystrophy. Cell.

[11] Chang WJ, Iannaccone ST, Lau KS, Masters BS, McCabe TJ, McMillan K, Padre RC, Spencer MJ, Tidball JG, Stull JT (1996). Neuronal nitric oxide synthase and dystrophin-deficient muscular dystrophy. Proc Natl Acad Sci.

[12] Leary SC, Battersby BJ, Hansford RG, Moyes CD (1998). Interactions between bioenergetics and mitochondrial biogenesis. Biochim Biophys Acta.

[13] Thomas GD, Sander M, Lau KS, Huang PL, Stull JT, Victor RG (1998). Impaired metabolic modulation of α-adrenergic vasoconstriction in dystrophin-deficient skeletal muscle. Proc Natl Acad Sci.

[14] Vaghy PL, Fang J, Wu W, Vaghy LP (1998). Increased caveolin-3 levels in mdx mouse muscles. FEBS Lett.

[15] Judge LM, Haraguchiln M, Chamberlain JS (2006). Dissecting the signaling and mechanical functions of the dystrophin-glycoprotein complex. J Cell Sci.

[16] Gücüyener K, Ergenekon E, Erbas D, Pinarli G, Serdaroğlu A (2000). The serum nitric oxide levels in patients with Duchenne muscular dystrophy. Brain and Development.

[17] Kasai T, Abeyama K, Hashiguchi T, Fukunaga H, Osame M, Maruyama K (2004). Decreased total nitric oxide production in patients with Duchenne muscular dystrophy. J Biomed Sci.

[18] Barton ER, Morris L, Kawana M, Bish LT, Toursel T (2005). Systemic administration of L-arginine benefits mdx skeletal muscle function. Muscle Nerve.

[19] McConell GK, Rattigan S, Lee-Young RS, Wadley GD, Merry TL (2012). Skeletal muscle nitric oxide signaling and exercise: a focus on glucose metabolism. Am J Physiol Endocrinol Metab.

[20] Wehling-Henricks M, Oltmann M, Rinaldi C, Myung KH, Tidball JG (2009). Loss of positive allosteric interactions between neuronal nitric oxide synthase and phosphofructokinase contributes to defects in glycolysis and increased fatigability in muscular dystrophy. Hum Mol Genet.

[21] Frascarelli M, Rocchi L, Feola I (1988). EMG computerized analysis of localized fatigue in Duchenne muscular dystrophy. Muscle Nerve.

[22] Wineinger MA, Walsh SA, Abresch RT (1998). The effect of age and temperature on mdx muscle fatigue. Muscle Nerve.

[23] Vignos PJ, Lefkowitz M (1959). A biochemical study of certain skeletal muscle constituents in human progressive muscular dystrophy. J Clin Investig.

[24] Chi MMY, Hintz CS, McKee D, Felder S, Grant N, Kaiser KK, Lowry OH (1987). Effect of Duchenne muscular dystrophy on enzymes of energy metabolism in individual muscle fibers. Metabolism.

[25] Austin L, De Niese M, McGregor A, Arthur H, Gurusinghe A, Gould M (1992). Potential oxyradical damage and energy status in individual muscle fibres from degenerating muscle diseases. Neuromuscul Disord.

[26] Cole M, Rafael J, Taylor D, Lodi R, Davies K, Styles P (2002). A quantitative study of bioenergetics in skeletal muscle lacking utrophin and dystrophin. Neuromuscul Disord.

[27] Buglioni A, Burnett Jr JC. New Pharmacological Strategies to Increase cGMP. Annu Rev Med. 2016;67:229-43. 10.1146/annurev-med-052914-091923 26473417

[28] Treuer AV, Gonzalez DR (2015). Nitric oxide synthases, S-nitrosylation and cardiovascular health: from molecular mechanisms to therapeutic opportunities (review). Mol Med Rep.

[29] Chaubourt E, Fossier P, Baux G, Leprince C, Israël M, De La Porte S (1999). Nitric oxide and l-arginine cause an accumulation of utrophin at the sarcolemma: a possible compensation for dystrophin loss in Duchenne muscular dystrophy. Neurobiol Dis.

[30] Voisin V, Sebrie C, Matecki S, Yu H, Gillet B, Ramonatxo M, Israel M, De la Porte S (2005). L-arginine improves dystrophic phenotype in mdx mice. Neurobiol Dis.

[31] Hnia K, Gayraud J, Hugon G, Ramonatxo M, De La Porte S, Matecki S, Mornet D (2008). L-arginine decreases inflammation and modulates the nuclear factor-κB/matrix metalloproteinase cascade in mdx muscle fibers. Am J Pathol.

[32] Vianello S, Yu H, Voisin V, Haddad H, He X, Foutz AS, Sebrié C, Gillet B, Roulot M, Fougerousse F (2013). Arginine butyrate: a therapeutic candidate for Duchenne muscular dystrophy. FASEB J.

[33] Vianello S, Consolaro F, Bich C, Cancela J-M, Roulot M, Lanchec E, Touboul D, Brunelle A, Israël M, Benoit E, de la Porte S (2014). Low doses of arginine butyrate derivatives improve dystrophic phenotype and restore membrane integrity in DMD models. FASEB J.

[34] Archer JD, Vargas CC, Anderson JE: Persistent and improved functional gain in mdx dystrophic mice after treatment with L-arginine and deflazacort. FASEB J*.* 2006. 10.1096/fj.05-4821fje 16464957

[35] Hafner P, Bonati U, Erne B, Schmid M, Rubino D, Pohlman U, Peters T, Rutz E, Frank S, Neuhaus C (2015). Improved muscle function in duchenne muscular dystrophy through L-arginine and metformin: an investigator-initiated, open-label, single-center, proof-of-concept-study. PloS one.

[36] Hafner P, Bonati U, Rubino D, Gocheva V, Zumbrunn T, Gueven N, Fischer D (2016). Treatment with L-citrulline and metformin in Duchenne muscular dystrophy: study protocol for a single-centre, randomised, placebo-controlled trial. Trials.

[37] Wijnands KA, Vink H, Briedé JJ, Van Faassen EE, Lamers WH, Buurman WA, Poeze M (2012). Citrulline a more suitable substrate than arginine to restore NO production and the microcirculation during endotoxemia. PloS one.

[38] Guerron AD, Rawat R, Sali A, Spurney CF, Pistilli E, Cha H-J, Pandey GS, Gernapudi R, Francia D, Farajian V (2010). Functional and molecular effects of arginine butyrate and prednisone on muscle and heart in the mdx mouse model of Duchenne muscular dystrophy. PloS one.

[39] Böger RH, Bode-Böger SM (2001). The clinical pharmacology of L-arginine. Annu Rev Pharmacol Toxicol.

[40] Boca SM, Nishida M, Harris M, Rao S, Cheema AK, Gill K, Seol H, Morgenroth LP, Henricson E, McDonald C (2016). Discovery of metabolic biomarkers for duchenne muscular dystrophy within a natural history study. PloS one.

[41] Ramachandran J, Schneider JS, Crassous PA, Zheng R, Gonzalez JP, Xie LH, Beuve A, Fraidenraich D, Peluffo RD (2013). Nitric oxide signalling pathway in Duchenne muscular dystrophy mice: up-regulation of L-arginine transporters. Biochem J.

[42] Wehling M, Spencer MJ, Tidball JG (2001). A nitric oxide synthase transgene ameliorates muscular dystrophy in mdx mice. J Cell Biol.

[43] Nguyen HX, Tidball JG (2003). Expression of a muscle‐specific, nitric oxide synthase transgene prevents muscle membrane injury and reduces muscle inflammation during modified muscle use in mice. J Physiol.

[44] Wehling-Henricks M, Jordan MC, Roos KP, Deng B, Tidball JG (2005). Cardiomyopathy in dystrophin-deficient hearts is prevented by expression of a neuronal nitric oxide synthase transgene in the myocardium. Hum Mol Genet.

[45] Shiao T, Fond A, Deng B, Wehling-Henricks M, Adams ME, Froehner SC, Tidball JG (2004). Defects in neuromuscular junction structure in dystrophic muscle are corrected by expression of a NOS transgene in dystrophin-deficient muscles, but not in muscles lacking α-and β1-syntrophins. Hum Mol Genet.

[46] Wehling-Henricks M, Tidball JG (2011). Neuronal nitric oxide synthase-rescue of dystrophin/utrophin double knockout mice does not require nNOS localization to the cell membrane. PLoS One.

[47] Tidball JG, Wehling-Henricks M (2004). Expression of a NOS transgene in dystrophin-deficient muscle reduces muscle membrane damage without increasing the expression of membrane-associated cytoskeletal proteins. Mol Genet Metab.

[48] Rebolledo DL, Kim MJ, Whitehead NP, Adams ME, Froehner SC. Sarcolemmal targeting of nNOSμ improves contractile function of mdx muscle. Hum Mol Genet. 2016;25(1):158-66. 10.1093/hmg/ddv466 PMC4690500 26604149

[49] Pons F, Robert A, Marini J, Leger J (1994). Does utrophin expression in muscles of mdx mice during postnatal development functionally compensate for dystrophin deficiency?. J Neurol Sci.

[50] Zhang Y, Duan D (2011). Novel mini–dystrophin gene dual adeno-associated virus vectors restore neuronal nitric oxide synthase expression at the sarcolemma. Hum Gene Ther.

[51] Lai Y, Thomas GD, Yue Y, Yang HT, Li D, Long C, Judge L, Bostick B, Chamberlain JS, Terjung RL (2009). Dystrophins carrying spectrin-like repeats 16 and 17 anchor nNOS to the sarcolemma and enhance exercise performance in a mouse model of muscular dystrophy. J Clin Invest.

[52] Zhang Y, Yue Y, Li L, Hakim CH, Zhang K, Thomas GD, Duan D (2013). Dual AAV therapy ameliorates exercise-induced muscle injury and functional ischemia in murine models of Duchenne muscular dystrophy. Hum Mol Genet.

[53] Laine R, de Montellano PR (1998). Neuronal nitric oxide synthase isoforms alpha and mu are closely related calpain-sensitive proteins. Mol Pharmacol.

[54] Kumamoto T, Ueyama H, Watanabe S, Yoshioka K, Miike T, Goll DE, Ando M, Tsuda T (1995). Immunohistochemical study of calpain and its endogenous inhibitor in the skeletal muscle of muscular dystrophy. Acta Neuropathol.

[55] Gonzalez DR, Beigi F, Treuer AV, Hare JM (2007). Deficient ryanodine receptor S-nitrosylation increases sarcoplasmic reticulum calcium leak and arrhythmogenesis in cardiomyocytes. Proc Natl Acad Sci.

[56] Soderling SH, Beavo JA (2000). Regulation of cAMP and cGMP signaling: new phosphodiesterases and new functions. Curr Opin Cell Biol.

[57] Wallis RM, Corbin JD, Francis SH, Ellis P (1999). Tissue distribution of phosphodiesterase families and the effects of sildenafil on tissue cyclic nucleotides, platelet function, and the contractile responses of trabeculae carneae and aortic rings in vitro. Am J Cardiol.

[58] Bloom TJ (2002). Cyclic nucleotide phosphodiesterase isozymes expressed in mouse skeletal muscle. Can J Physiol Pharmacol.

[59] Senzaki H, Smith CJ, Juang GJ, Isoda T, Mayer SP, Ohler A, Paolocci N, Tomaselli GF, Hare JM, Kass DA (2001). Cardiac phosphodiesterase 5 (cGMP-specific) modulates β-adrenergic signaling in vivo and is down-regulated in heart failure. FASEB J.

[60] Asai A, Sahani N, Kaneki M, Ouchi Y, Martyn JJ, Yasuhara SE (2007). Primary role of functional ischemia, quantitative evidence for the two-hit mechanism, and phosphodiesterase-5 inhibitor therapy in mouse muscular dystrophy. PLoS One.

[61] De Arcangelis V, Strimpakos G, Gabanella F, Corbi N, Luvisetto S, Magrelli A, Onori A, Passananti C, Pisani C, Rome S (2016). Pathways implicated in tadalafil amelioration of duchenne muscular dystrophy. J Cell Physiol.

[62] Kobayashi YM, Rader EP, Crawford RW, Iyengar NK, Thedens DR, Faulkner JA, Parikh SV, Weiss RM, Chamberlain JS, Moore SA (2008). Sarcolemma-localized nNOS is required to maintain activity after mild exercise. Nature.

[63] Nelson MD, Rader F, Tang X, Tavyev J, Nelson SF, Miceli MC, Elashoff RM, Sweeney HL, Victor RG (2014). PDE5 inhibition alleviates functional muscle ischemia in boys with Duchenne muscular dystrophy. Neurology.

[64] Martin EA, Barresi R, Byrne BJ, Tsimerinov EI, Scott BL, Walker AE, Gurudevan SV, Anene F, Elashoff RM, Thomas GD (2012). Tadalafil alleviates muscle ischemia in patients with Becker muscular dystrophy. Sci Transl Med.

[65] Hammers DW, Sleeper MM, Forbes SC, Shima A, Walter GA, Sweeney HL (2016). Tadalafil treatment delays the onset of cardiomyopathy in dystrophin‐deficient hearts. J Am Heart Assoc.

[66] Percival JM, Whitehead NP, Adams ME, Adamo CM, Beavo JA, Froehner SC (2012). Sildenafil reduces respiratory muscle weakness and fibrosis in the mdx mouse model of Duchenne muscular dystrophy. J Pathol.

[67] Percival JM, Siegel MP, Knowels G, Marcinek DJ (2013). Defects in mitochondrial localization and ATP synthesis in the mdx mouse model of Duchenne muscular dystrophy are not alleviated by PDE5 inhibition. Hum Mol Genet.

[68] Kawahara G, Karpf JA, Myers JA, Alexander MS, Guyon JR, Kunkel LM (2011). Drug screening in a zebrafish model of Duchenne muscular dystrophy. Proc Natl Acad Sci.

[69] Kawahara G, Gasperini MJ, Myers JA, Widrick JJ, Eran A, Serafini PR, Alexander MS, Pletcher MT, Morris CA, Kunkel LM. Dystrophic muscle improvement in zebrafish via increased heme oxygenase signaling. Hum Mol Genet. 2013:ddt579. 10.1093/hmg/ddt579 PMC3943523 24234649

[70] Khairallah M, Khairallah R, Young M, Allen B, Gillis M, Danialou G, Deschepper C, Petrof B, Des Rosiers C (2008). Sildenafil and cardiomyocyte-specific cGMP signaling prevent cardiomyopathic changes associated with dystrophin deficiency. Proc Natl Acad Sci.

[71] Adamo CM, Dai D-F, Percival JM, Minami E, Willis MS, Patrucco E, Froehner SC, Beavo JA (2010). Sildenafil reverses cardiac dysfunction in the mdx mouse model of Duchenne muscular dystrophy. Proc Natl Acad Sci.

[72] Leung DG, Herzka DA, Thompson WR, He B, Bibat G, Tennekoon G, Russell SD, Schuleri KH, Lardo AC, Kass DA (2014). Sildenafil does not improve cardiomyopathy in Duchenne/Becker muscular dystrophy. Ann Neurol.

[73] Witting N, Kruuse C, Nyhuus B, Prahm KP, Citirak G, Lundgaard SJ, Huth S, Vejlstrup N, Lindberg U, Krag TO (2014). Effect of sildenafil on skeletal and cardiac muscle in Becker muscular dystrophy. Ann Neurol.

[74] Wang G, Lu Q (2013). A nitrate ester of sedative alkyl alcohol improves muscle function and structure in a murine model of Duchenne muscular dystrophy. Mol Pharm.

[75] Miglietta D, De Palma C, Sciorati C, Vergani B, Pisa V, Villa A, Ongini E, Clementi E (2015). Naproxcinod shows significant advantages over naproxen in the mdx model of Duchenne muscular dystrophy. Orphanet J Rare Dis.

[76] Uaesoontrachoon K, Quinn JL, Tatem KS, Van Der Meulen JH, Yu Q, Phadke A, Miller BK, Gordish-Dressman H, Ongini E, Miglietta D. Long-term treatment with naproxcinod significantly improves skeletal and cardiac disease phenotype in the mdx mouse model of dystrophy. Hum Mol Genet 2014:ddu033. 10.1093/hmg/ddu033 PMC4030778 24463621

[77] Thomas GD, Ye J, De Nardi C, Monopoli A, Ongini E, Victor RG (2012). Treatment with a nitric oxide-donating NSAID alleviates functional muscle ischemia in the mouse model of Duchenne muscular dystrophy. PLoS ONE.

[78] Brunelli S, Sciorati C, D’Antona G, Innocenzi A, Covarello D, Galvez BG, Perrotta C, Monopoli A, Sanvito F, Bottinelli R (2007). Nitric oxide release combined with nonsteroidal antiinflammatory activity prevents muscular dystrophy pathology and enhances stem cell therapy. Proc Natl Acad Sci.

[79] Sciorati C, Staszewsky L, Zambelli V, Russo I, Salio M, Novelli D, Di Grigoli G, Moresco RM, Clementi E, Latini R (2013). Ibuprofen plus isosorbide dinitrate treatment in the mdx mice ameliorates dystrophic heart structure. Pharmacol Res.

[80] Mizunoya W, Upadhaya R, Burczynski FJ, Wang G, Anderson JE (2011). Nitric oxide donors improve prednisone effects on muscular dystrophy in the mdx mouse diaphragm. Am J Phys Cell Phys.

[81] D’Angelo MG, Gandossini S, Boneschi FM, Sciorati C, Bonato S, Brighina E, Comi GP, Turconi AC, Magri F, Stefanoni G (2012). Nitric oxide donor and non steroidal anti inflammatory drugs as a therapy for muscular dystrophies: evidence from a safety study with pilot efficacy measures in adult dystrophic patients. Pharmacol Res.

[82] Pacher P, Beckman JS, Liaudet L (2007). Nitric oxide and peroxynitrite in health and disease. Physiol Rev.

[83] Hart J, Dulhunty A (2000). Nitric oxide activates or inhibits skeletal muscle ryanodine receptors depending on its concentration, membrane potential and ligand binding. J Membr Biol.

[84] Gangolli SD, Van Den Brandt PA, Feron VJ, Janzowsky C, Koeman JH, Speijers GJ, Spiegelhalder B, Walker R, Wishnok JS (1994). Nitrate, nitrite and N-nitroso compounds. Eur J Pharmacol.

[85] Hord NG, Tang Y, Bryan NS (2009). Food sources of nitrates and nitrites: the physiologic context for potential health benefits. Am J Clin Nutr.

[86] Doel JJ, Benjamin N, Hector MP, Rogers M, Allaker RP (2005). Evaluation of bacterial nitrate reduction in the human oral cavity. Eur J Oral Sci.

[87] Kim-Shapiro DB, Gladwin MT (2014). Mechanisms of nitrite bioactivation. Nitric Oxide.

[88] Lundberg JO, Weitzberg E, Gladwin MT (2008). The nitrate–nitrite–nitric oxide pathway in physiology and therapeutics. Nat Rev Drug Discov.

[89] Larsen FJ, Schiffer TA, Borniquel S, Sahlin K, Ekblom B, Lundberg JO, Weitzberg E (2011). Dietary inorganic nitrate improves mitochondrial efficiency in humans. Cell Metab.

[90] Hernández A, Schiffer TA, Ivarsson N, Cheng AJ, Bruton JD, Lundberg JO, Weitzberg E, Westerblad H (2012). Dietary nitrate increases tetanic [Ca2+] i and contractile force in mouse fast‐twitch muscle. J Physiol.

[91] Haider G, Folland JP (2014). Nitrate supplementation enhances the contractile properties of human skeletal muscle. Med Sci Sports Exerc.

[92] Lansley KE, Winyard PG, Fulford J, Vanhatalo A, Bailey SJ, Blackwell JR, DiMenna FJ, Gilchrist M, Benjamin N, Jones AM (2011). Dietary nitrate supplementation reduces the O2 cost of walking and running: a placebo-controlled study. J Appl Physiol.

[93] Cermak NM, Res P, Stinkens R, Lundberg JO, Gibala MJ, van Loon LJ (2012). No improvement in endurance performance after a single dose of beetroot juice. Int J Sport Nutr ExercMetab.

[94] Bond V, Curry BH, Adams RG, Millis RM, Haddad GE (2013). Cardiorespiratory function associated with dietary nitrate supplementation. Appl Physiol Nutr Metab.

[95] Whitfield J, Ludzki A, Heigenhauser G, Senden J, Verdijk L, Loon L, Spriet L, Holloway G (2016). Beetroot juice supplementation reduces whole body oxygen consumption but does not improve indices of mitochondrial efficiency in human skeletal muscle. J Physiol.

[96] Bailey SJ, Winyard P, Vanhatalo A, Blackwell JR, DiMenna FJ, Wilkerson DP, Tarr J, Benjamin N, Jones AM (2009). Dietary nitrate supplementation reduces the O2 cost of low-intensity exercise and enhances tolerance to high-intensity exercise in humans. J Appl Physiol.

[97] Bond H, Morton L, Braakhuis AJ (2012). Dietary nitrate supplementation improves rowing performance in well-trained rowers.

[98] Hoon MW, Fornusek C, Chapman PG, Johnson NA (2015). The effect of nitrate supplementation on muscle contraction in healthy adults. Eur J Sport Sci.

[99] Aucouturier J, Boissière J, Pawlak-Chaouch M, Cuvelier G, Gamelin F-X (2015). Effect of dietary nitrate supplementation on tolerance to supramaximal intensity intermittent exercise. Nitric Oxide.

[100] Cermak NM, Gibala MJ, Van Loon LJ (2012). Nitrate supplementation’s improvement of 10-km time-trial performance in trained cyclists. Int J Sport Nutr Exerc Metab.

[101] Lansley KE, Winyard PG, Bailey SJ, Vanhatalo A, Wilkerson DP, Blackwell JR, Gilchrist M, Benjamin N, Jones AM (2011). Acute dietary nitrate supplementation improves cycling time trial performance. Med Sci Sports Exerc.

[102] Ashmore T, Roberts LD, Morash AJ, Kotwica AO, Finnerty J, West JA, Murfitt SA, Fernandez BO, Branco C, Cowburn AS (2015). Nitrate enhances skeletal muscle fatty acid oxidation via a nitric oxide-cGMP-PPAR-mediated mechanism. BMC Biol.

[103] Ashmore T, Fernandez BO, Branco‐Price C, West JA, Cowburn AS, Heather LC, Griffin JL, Johnson RS, Feelisch M, Murray AJ (2014). Dietary nitrate increases arginine availability and protects mitochondrial complex I and energetics in the hypoxic rat heart. J Physiol.

[104] Berry MJ, Justus NW, Hauser JI, Case AH, Helms CC, Basu S, Rogers Z, Lewis MT, Miller GD (2015). Dietary nitrate supplementation improves exercise performance and decreases blood pressure in COPD patients. Nitric Oxide.

[105] Kerley CP, Cahill K, Bolger K, McGowan A, Burke C, Faul J, Cormican L (2015). Dietary nitrate supplementation in COPD: An acute, double-blind, randomized, placebo-controlled, crossover trial☆. Nitric Oxide.

[106] Kenjale AA, Ham KL, Stabler T, Robbins JL, Johnson JL, VanBruggen M, Privette G, Yim E, Kraus WE, Allen JD (2011). Dietary nitrate supplementation enhances exercise performance in peripheral arterial disease. J Appl Physiol.

[107] Timpani CA, Trewin AJ, Stojanovska V, Robinson A, Goodman CA, Nurgali K, Betik AC, Stepto N, Hayes A, McConell GK. Attempting to compensate for reduced neuronal nitric oxide synthase protein with nitrate supplementation cannot overcome metabolic dysfunction but rather Has detrimental effects in dystrophin-deficient mdx muscle. Neurotherapeutics. 2016;1–18. 10.1007/s13311-016-0494-7 PMC5398978 27921261

[108] Carlström M, Larsen FJ, Nyström T, Hezel M, Borniquel S, Weitzberg E, Lundberg JO (2010). Dietary inorganic nitrate reverses features of metabolic syndrome in endothelial nitric oxide synthase-deficient mice. Proc Natl Acad Sci.

[109] DePirro R, Lauro R, Testa I, Ferretti I, De Martinis C, Dellatonio R (1982). Decreased insulin receptors but normal glucose metabolism in Duchenne muscular dystrophy. Science.

[110] Kuznetsov AV, Winkler K, Wiedemann F, von Bossanyi P, Dietzmann K, Kunz WS (1998). Impaired mitochondrial oxidative phosphorylation in skeletal muscle of the dystrophin-deficient mdx mouse. Mol Cell Biochem.

[111] Passaquin AC, Renard M, Kay L, Challet C, Mokhtarian A, Wallimann T, Ruegg UT (2002). Creatine supplementation reduces skeletal muscle degeneration and enhances mitochondrial function in mdx mice. Neuromuscul Disord.

[112] Aquilano K, Baldelli S, Ciriolo MR (2014). Nuclear recruitment of neuronal nitric-oxide synthase by α-syntrophin is crucial for the induction of mitochondrial biogenesis. J Biol Chem.

[113] Timpani CA, Trewin, Adam J, Stojanovska, Vanesa, Robinson, Ainsley, Goodman, Craig A, Nurgali, Kulmira, Betik, Andrew C, Stepto, Nigel, Hayes, Alan, McConell, Glenn K, Rybalka, Emma Attempting to compensate for reduced nNOS protein with nitrate supplementation cannot overcome metabolic dysfunction but rather has detrimental effects in dystrophin-deficient mdx muscle. Neurotherapeutics. 2016. 10.1007/s13311-016-0494-7 PMC5398978 27921261

[114] Uaesoontrachoon K, Quinn JL, Tatem KS, Van Der Meulen JH, Yu Q, Phadke A, Miller BK, Gordish-Dressman H, Ongini E, Miglietta D, Nagaraju K (2014). Long-term treatment with naproxcinod significantly improves skeletal and cardiac disease phenotype in the mdx mouse model of dystrophy. Hum Mol Genet.

[115] Nelson MD, Rosenberry R, Barresi R, Tsimerinov EI, Rader F, Tang X, Mason ON, Schwartz A, Stabler T, Shidban S (2015). Sodium nitrate alleviates functional muscle ischaemia in patients with Becker muscular dystrophy. J Physiol.

